# Naringin Attenuates Diabetic Neuropathic Pain and Modulates TRPV1-Related Ca^2+^ Dysregulation and MAPK/NF-ΚB Signaling

**DOI:** 10.3390/ph19101613

**Published:** 2026-10-10

**Authors:** Xiaoyi Qiu, Lisha Chen, Ting Han, Hongji Wang, Juping Xing, Jiayi Zheng, Changshui Xu

**Affiliations:** 1 School of Clinical Medicine, Jiangxi Medical College, Shangrao 334000, China; qiuxy1998@outlook.com (X.Q.); hanting02626@outlook.com (T.H.);; 2 Department of Physiology, Basic Medical College, Nanchang University, Nanchang 330006, China; rati7577@outlook.com (L.C.);

**Keywords:** transient receptor potential vanilloid 1, naringin, diabetic neuropathic pain, dorsal root ganglion

## Abstract

**Background/Objectives:** Diabetic neuropathic pain (DNP) is a common complication of diabetes characterized by sensory abnormalities, including mechanical allodynia and thermal hyperalgesia. This study aimed to investigate the effects of naringin on experimental DNP and evaluate its association with dorsal root ganglion (DRG) neuronal injury, TRPV1-related calcium imbalance, and inflammatory signaling. **Methods:** Primary rat DRG neurons were exposed to high-glucose/high-free-fatty-acid (HGHF) conditions (50 mM glucose and 0.60 mM free fatty acids) and treated with naringin (40 or 80 μM). Cell viability, apoptosis, intracellular Ca^2+^ levels, gene/protein expression, and signaling changes were assessed using CCK-8 assay, Annexin V-FITC/PI flow cytometry, Fluo-4 AM imaging, qRT-PCR, and Western blotting. Male Sprague–Dawley rats were fed a high-fat/high-sucrose diet for 4 weeks, injected with streptozotocin (35 mg kg^−1^), and treated with naringin (50 mg kg^−1^ day^−1^) by gavage for 2 weeks. **Results:** HGHF exposure increased neuronal injury, TRPV1 expression, intracellular Ca^2+^ accumulation, and p38 MAPK/NF-κB signaling activation. Naringin attenuated these changes in vitro, with stronger effects observed at 80 μM. In DNP rats, naringin improved pain-related behaviors, partially alleviated metabolic abnormalities, reduced TRPV1 expression and serum IL-1β/TNF-α levels, and decreased phosphorylation of p38 MAPK, NF-κB p65, and ERK in DRGs. **Conclusions:** Naringin alleviated experimental DNP-associated behavioral and molecular abnormalities, suggesting potential protective effects involving neuronal injury, calcium regulation, and inflammatory responses.

## 1. Introduction

Diabetes mellitus represents a rapidly growing global health burden. According to the 11th edition of the International Diabetes Federation Diabetes Atlas, approximately 589 million adults aged 20–79 years were living with diabetes in 2024, corresponding to 11.1% of the global adult population, and this number is projected to increase to approximately 853 million by 2050 [1]. Diabetic peripheral neuropathy (DPN) is among the most prevalent chronic complications of diabetes and is associated with substantial morbidity, functional impairment, and reduced quality of life [2]. Its pathogenesis is multifactorial and involves hyperglycemia, dyslipidemia, mitochondrial dysfunction, oxidative stress, microvascular abnormalities, and chronic inflammation, which collectively contribute to progressive peripheral nerve injury [3,4,5]. Recent evidence further emphasizes the close interaction between metabolic dysregulation and redox imbalance in diabetes [6,7,8]. In painful diabetic neuropathy, contemporary mechanistic analyses highlight the convergence of metabolic stress, mitochondrial dysfunction, ion-channel dysregulation, neuroinflammation, and nociceptor sensitization in the development and persistence of pain [9]. A clinically important subgroup develops painful diabetic neuropathy, referred to as diabetic neuropathic pain (DNP) in the present study, with manifestations that include spontaneous pain, burning sensations, mechanical allodynia, and thermal hyperalgesia [10,11]. Current pharmacological management is directed largely toward symptomatic pain relief, and treatment responses are often incomplete, whereas effective disease-modifying strategies capable of preventing or reversing diabetes-associated neural injury remain limited [12,13]. Therefore, therapeutic approaches that can reduce nociceptive hypersensitivity while also protecting peripheral sensory neurons remain of substantial interest.

Dorsal root ganglion (DRG) neurons are primary sensory neurons that transmit peripheral nociceptive information and are vulnerable to diabetes-associated metabolic and inflammatory stress [3,4]. Transient receptor potential vanilloid 1 (TRPV1) is a polymodal, non-selective cation channel expressed predominantly in nociceptive sensory neurons and can be activated or sensitized by capsaicin, noxious heat, acidic conditions, and inflammatory mediators [14,15,16]. Altered TRPV1 expression and sensitization have been linked to nociceptor hyperexcitability and persistent pain [15,17], and TRPV1 has been implicated in diabetic neuropathic pain [18,19]. Human studies have also reported increased TRPV1-positive cutaneous innervation in patients with painful diabetic polyneuropathy and ongoing burning pain [20]. Recent literature continues to identify TRPV1 as a therapeutically relevant interface between sensory nerve activity, nociceptive signaling, inflammation, and metabolic regulation in diabetic neuropathy [21]. Because TRPV1 activation permits Ca^2+^ influx, altered TRPV1 activity may be accompanied by disturbances in intracellular Ca^2+^ homeostasis and sensory neuronal excitability [16].

Inflammatory and stress-responsive signaling pathways also contribute to neuropathic pain. MAPKs, particularly p38 MAPK and ERK, participate in neuronal sensitization, inflammatory responses, and pain hypersensitivity [22,23], whereas NF-κB coordinates inflammatory gene expression and cytokine production under metabolic and immune stress. Experimental evidence supports functional associations among TRPV1, p38 MAPK, NF-κB, and inflammatory signaling in diabetic neuropathic pain [24]. However, these pathways form an interconnected and potentially reciprocal regulatory network rather than a universally defined linear cascade. Accordingly, concurrent changes in TRPV1, intracellular Ca^2+^, MAPK signaling, and NF-κB activity should not be interpreted as evidence of a fixed causal hierarchy in the absence of pathway-specific intervention.

Naringin is a naturally occurring flavanone glycoside abundant in citrus fruits and has been reported to exert antioxidant, anti-inflammatory, anti-apoptotic, metabolic regulatory, and neuroprotective effects [25,26,27,28]. It has also shown anti-nociceptive effects in experimental pain-related conditions [29]. Importantly, previous electrophysiological evidence demonstrated that naringin can directly inhibit capsaicin-evoked TRPV1 activity, providing a mechanistic basis for investigating its relationship with TRPV1-dependent nociceptive signaling [30]. More recently, naringin and its aglycone naringenin have also shown antinociceptive effects accompanied by altered TRPV1 expression in an experimental neuropathic pain model [31]. These diverse actions suggest that naringin is pharmacologically pleiotropic rather than a selective modulator of a single molecular target [25,27,28]. In addition, orally administered naringin undergoes extensive intestinal biotransformation, including conversion to naringenin and other microbial metabolites, which may contribute to its systemic biological effects [28,32]. Thus, the in vivo actions observed after oral naringin may reflect exposure to the parent compound, its metabolites, or indirect systemic effects.

Previous studies have shown that naringin can attenuate experimental diabetic neuropathy and improve metabolic, oxidative, inflammatory, and neural abnormalities in diabetic animals [27,33]. More recently, naringin was reported to reduce thermal and mechanical pain hypersensitivity in an STZ-based rat model of diabetic peripheral neuropathy, together with improvements in glycemic status, inflammatory cytokines, and oxidative-stress indices [34]. However, the relationship between these anti-nociceptive effects and DRG-specific TRPV1 expression, intracellular Ca^2+^ dysregulation, and MAPK/NF-κB-associated signaling remains incompletely characterized. The present study therefore used complementary in vitro and in vivo models to evaluate whether naringin attenuates DRG neuronal injury and pain-related phenotypes under diabetic metabolic stress and whether these effects are accompanied by coordinated changes in TRPV1 expression, intracellular Ca^2+^, MAPK/NF-κB-associated signaling, and inflammatory mediators.

## 2. Results

### 2.1. Naringin Attenuates HGHF-Induced DRG Neuronal Injury and Apoptosis In Vitro

**Figure 1:**
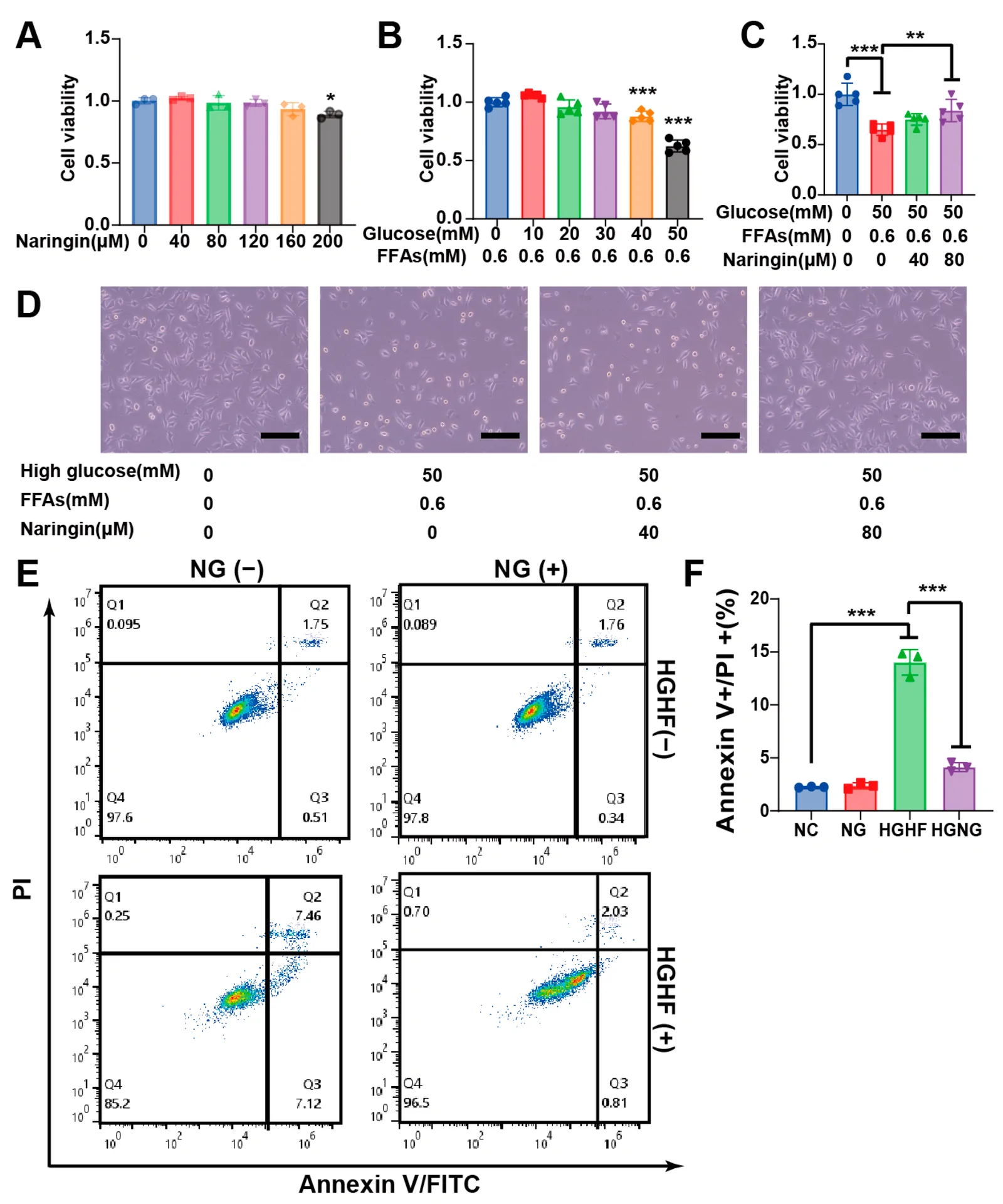
Naringin attenuates HGHF-induced injury and apoptosis in primary DRG neurons in vitro. (**A**) Cell viability of primary DRG neurons treated with naringin (40–200 μM) for 48 h to define the non-cytotoxic concentration range. (**B**) Effect of HGHF exposure (50 mM D-glucose + 0.60 mM FFAs) on DRG neuronal viability after 48 h. (**C**,**D**) Effects of 40 and 80 μM naringin on HGHF-associated cytotoxicity (scale bar = 100 μm, where applicable). (**E**) Representative Annexin V-FITC/PI flow-cytometry plots. (**F**) Quantification of total apoptotic cells (early + late apoptosis). Data are presented as mean ± SD from three independent experiments. Significant pairwise comparisons are indicated by brackets. Asterisks denote the corresponding significance levels (* *p* < 0.05, ** *p* < 0.01, and *** *p* < 0.001).

To define a non-cytotoxic concentration range, primary DRG neurons were exposed to naringin at 40–200 μM for 48 h. Cell viability remained comparable to untreated controls at 40–160 μM, whereas 200 μM naringin produced a modest but statistically significant reduction in viability, identifying 160 μM as the upper non-cytotoxic concentration under the conditions tested (Figure 1A).

Exposure to 50 mM D-glucose plus 0.60 mM FFAs for 48 h reduced DRG neuronal viability to 62.5 ± 5.0% of the control level (Figure 1B). Co-treatment with 40 or 80 μM naringin attenuated this reduction, with viability values of 75.0 ± 5.7% and 83.0 ± 11.0%, respectively (Figure 1C,D). The larger improvement observed at 80 μM supported the use of both concentrations for subsequent cellular analyses.

Flow-cytometric analysis further showed an increased proportion of apoptotic DRG neurons after HGHF exposure. Naringin co-treatment reduced the apoptotic proportion toward control levels (Figure 1E,F). Together, these findings indicate that naringin attenuated HGHF-associated loss of viability and apoptosis in primary DRG neurons.

### 2.2. Naringin Is Associated with Reduced TRPV1 Expression, Ca^2+^ Accumulation, and p38/NF-κB Activation in HGHF-Exposed DRG Neurons

**Figure 2:**
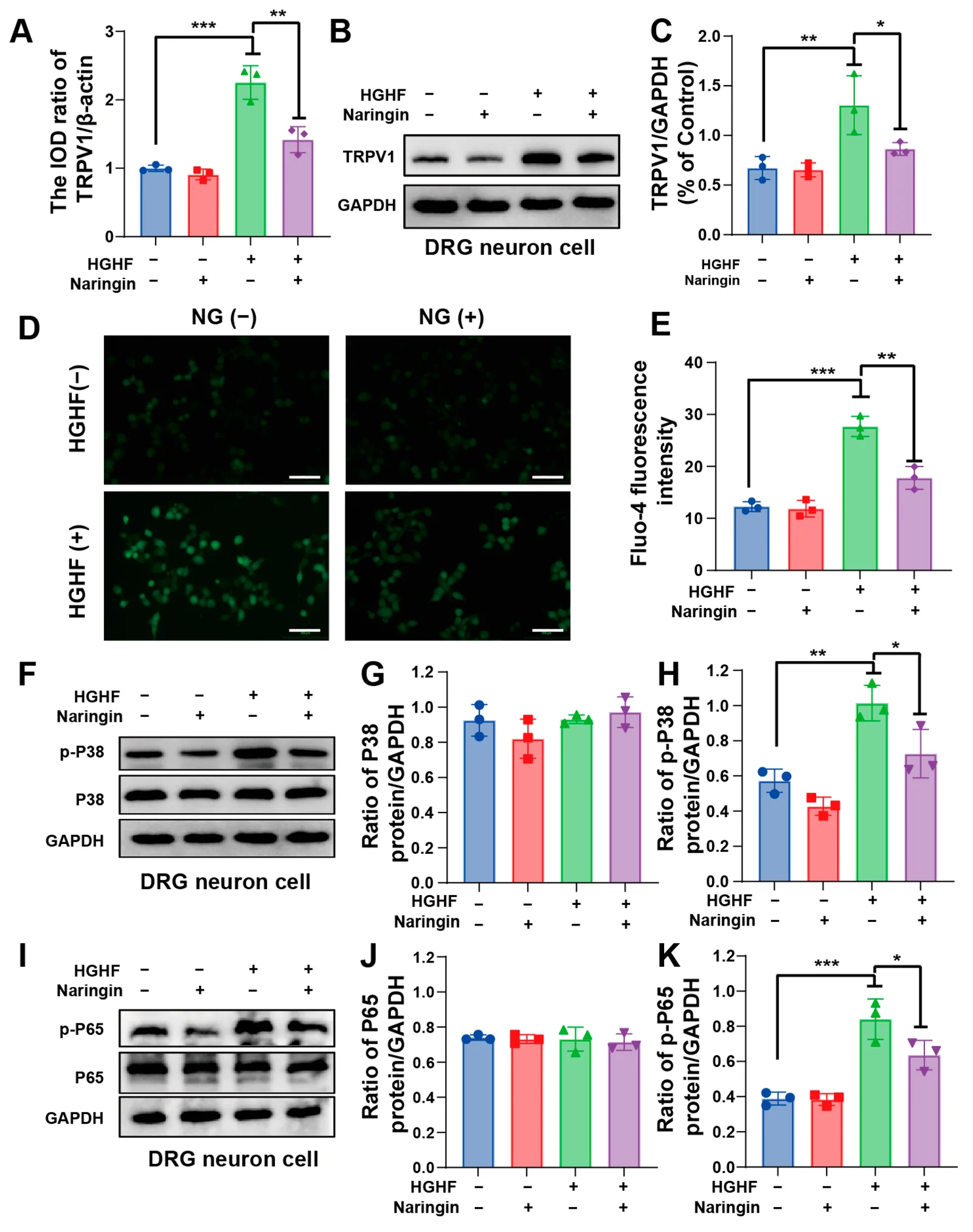
Naringin is associated with reduced TRPV1 expression, intracellular Ca^2+^ accumulation, and p38 MAPK/NF-κB activation in HGHF-exposed DRG neurons. (**A**) Relative TRPV1 mRNA expression measured by qRT-PCR and normalized to β-actin. (**B**) Representative Western blots of TRPV1. (**C**) Densitometric quantification of TRPV1 normalized to β-actin. (**D**) Representative Fluo-4 AM fluorescence images of intracellular Ca^2+^ (scale bar = 100 μm). (**E**) Experiment-level quantification of Fluo-4 fluorescence. (**F**–**H**) Representative p38/phospho-p38 blots and quantification of phospho-p38 normalized to total p38. (**I**–**K**) Representative NF-κB p65/phospho-p65 blots and quantification of phospho-p65 normalized to total p65. HGHF indicates 50 mM D-glucose + 0.60 mM FFAs for 48 h. qRT-PCR, Western blotting, and Ca^2+^ imaging were performed in three independent experiments per group. For Ca^2+^ imaging, at least five fields were examined in each experiment and a total of 50 neurons per treatment group were analyzed across the three experiments; individual neurons were observational subsamples and the independent experiment was the statistical unit. Data are presented as mean ± SD. Significant pairwise comparisons are indicated by brackets. Asterisks denote the corresponding significance levels (* *p* < 0.05, ** *p* < 0.01, and *** *p* < 0.001).

qRT-PCR and Western blot analyses showed that HGHF exposure increased TRPV1 mRNA and protein expression in DRG neurons, whereas both increases were attenuated by naringin co-treatment (Figure 2A–C).

In parallel, Fluo-4 AM imaging showed higher intracellular Ca^2+^ fluorescence after HGHF exposure. Naringin treatment reduced the elevated Ca^2+^ signal toward control levels (Figure 2D,E). Because the independent culture experiment served as the statistical unit, the 50 neurons analyzed per treatment group were treated as observational subsamples rather than independent biological replicates.

HGHF exposure was also accompanied by increased phosphorylation of p38 MAPK and NF-κB p65. Naringin co-treatment reduced p38 and p65 phosphorylation (Figure 2F–K). These data demonstrate coordinated changes in TRPV1 expression, intracellular Ca^2+^, and p38 MAPK/NF-κB-associated signaling after naringin treatment but do not establish a linear causal signaling cascade.

### 2.3. Naringin Improves Metabolic Measures and Attenuates Pain-Related Behaviors in Experimental DNP

**Figure 3:**
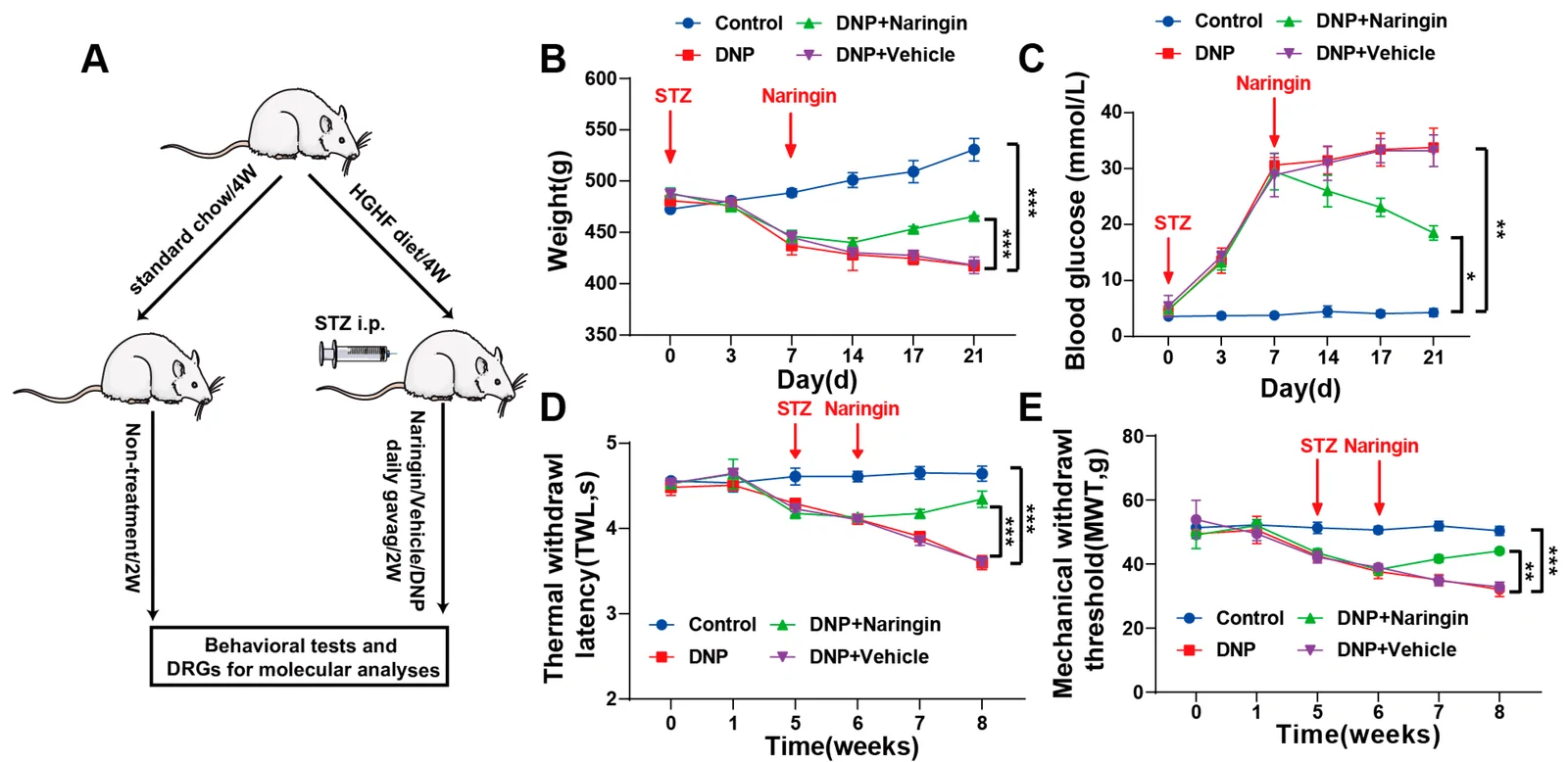
Naringin improves metabolic measures and attenuates pain-related behaviors in the HGHF/low-dose STZ rat model of DNP. (**A**) Experimental timeline for 4 weeks of HGHF dietary exposure, low-dose STZ administration, DNP assessment, naringin/vehicle treatment, and behavioral testing. (**B**) Body weight over time. (**C**) Fasting blood glucose over time. (**D**) Thermal withdrawal latency (TWL). (**E**) Mechanical withdrawal threshold (MWT). Data are presented as mean ± SD (*n* = 6 animals/group). Longitudinal outcomes were analyzed using two-way repeated-measures ANOVA with group and time as factors and evaluation of the group × time interaction, followed by Bonferroni-adjusted pairwise comparisons. Significant pairwise comparisons are indicated by brackets. Asterisks denote the corresponding significance levels (* *p* < 0.05, ** *p* < 0.01, and *** *p* < 0.001).

The in vivo DNP model was generated by 4 weeks of HGHF dietary exposure followed by low-dose STZ administration (Figure 3A). Diabetes was confirmed 72 h after STZ according to the prespecified glycemic criteria, and DNP was defined by a reduction in both MWT and TWL to <80% of the corresponding baseline values within 14 days after STZ administration. Compared with non-diabetic controls, DNP rats showed persistent hyperglycemia and lower body weight during the experimental period (Figure 3B,C).

During the 2-week treatment period, naringin partially attenuated the metabolic abnormalities, with higher body weight and lower fasting blood glucose than in vehicle-treated DNP rats (Figure 3B,C). Two-way repeated-measures ANOVA revealed significant effects of group, time, and group × time interaction for both metabolic outcomes. For body weight, significant effects were observed for group [F(3, 20) = 502.4, *p* < 0.0001], time [F(3.908, 78.16) = 177.4, *p* < 0.0001], and group × time interaction [F(15, 100) = 129.1, *p* < 0.0001]. For fasting blood glucose, significant effects were observed for group [F(3, 20) = 717.3, *p* < 0.0001], time [F(2.957, 59.14) = 691.3, *p* < 0.0001], and group × time interaction [F(15, 100) = 93.01, *p* < 0.0001]. Because these metabolic changes occurred in parallel with the behavioral and molecular improvements, the in vivo data do not distinguish direct neural actions of naringin from effects secondary to improved metabolic control.

By week 6, DNP rats displayed reduced thermal withdrawal latency (TWL) and mechanical withdrawal threshold (MWT), consistent with thermal hyperalgesia and mechanical allodynia (Figure 3D,E). Naringin increased both TWL and MWT relative to the Vehicle group during treatment, indicating attenuation of pain-related hypersensitivity. Two-way repeated-measures ANOVA revealed significant effects of group, time, and group × time interaction for both behavioral outcomes. For TWL, significant effects were observed for group [F(3, 20) = 484.5, *p* < 0.0001], time [F(3.730, 74.61) = 268.4, *p* < 0.0001], and group × time interaction [F(15, 100) = 73.30, *p* < 0.0001]. For MWT, significant effects were observed for group [F(3, 20) = 105.7, *p* < 0.0001], time [F(3.273, 65.47) = 219.4, *p* < 0.0001], and group × time interaction [F(15, 100) = 33.26, *p* < 0.0001]. Bonferroni-adjusted pairwise comparisons were used to evaluate between-group differences at the indicated time points (Figure 3D,E).

### 2.4. Naringin Is Associated with Reduced TRPV1 Expression and MAPK/NF-κB-Associated Inflammatory Signaling in DRGs of DNP Rats

**Figure 4:**
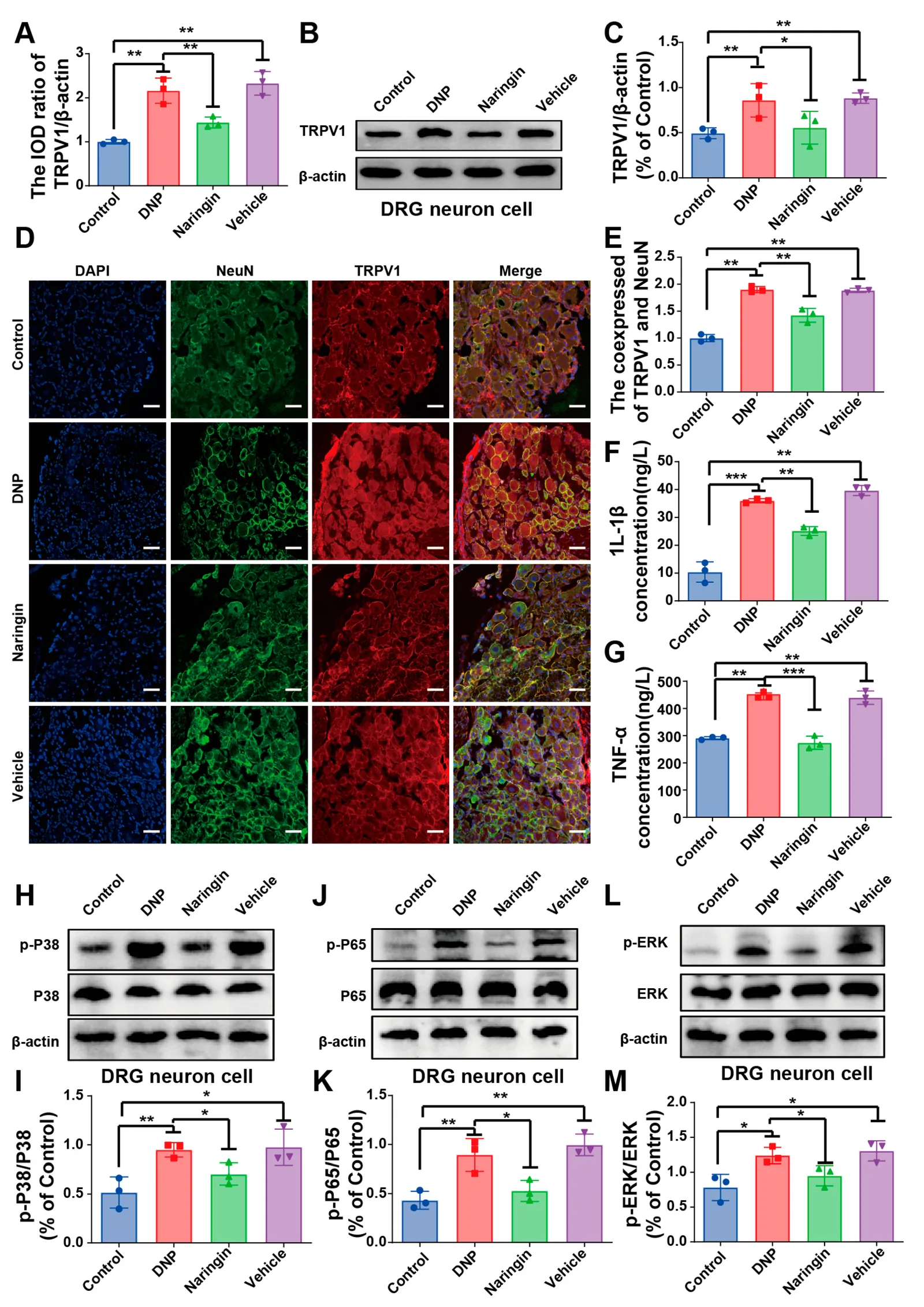
Naringin is associated with reduced TRPV1 expression and MAPK–NF-κB-associated inflammatory signaling in DRGs of DNP rats. (**A**) Relative TRPV1 mRNA expression in DRGs measured by qRT-PCR and normalized to β-actin. (**B**,**C**) Representative TRPV1 Western blots and densitometric quantification normalized to β-actin. (**D**) Representative immunofluorescence images of TRPV1 (red), NeuN (green), and DAPI (blue) in DRG sections (scale bar = 50 μm). (**E**) Quantification of TRPV1/NeuN co-localization using Pearson’s correlation coefficient. Immunofluorescence analysis included 3–4 animals per group, four sections per animal, and three non-overlapping fields per section. Field- and section-level measurements were averaged to obtain one value per animal, and the animal served as the statistical unit. (**F**,**G**) Serum IL-1β and TNF-α concentrations measured by ELISA. (**H**–**K**) Representative blots and densitometric quantification of phospho-p38/total p38 and phospho-NF-κB p65/total p65. (**L**,**M**) Representative blots and quantification of phospho-ERK/total ERK. For Western blotting, *n* = 3 independent biological replicates (animals) per group. Data are presented as mean ± SD. Significant pairwise comparisons are indicated by brackets. Asterisks denote the corresponding significance levels (* *p* < 0.05, ** *p* < 0.01, and *** *p* < 0.001).

TRPV1 mRNA expression was increased in DRGs from DNP rats compared with non-diabetic controls and was lower in naringin-treated animals (Figure 4A). Western blotting showed a similar pattern for TRPV1 protein expression (Figure 4B,C). These results indicate attenuation, rather than complete reversal, of diabetes-associated TRPV1 upregulation after naringin treatment.

Immunofluorescence staining showed greater TRPV1 signal within NeuN-positive DRG neurons in DNP rats and a lower TRPV1/NeuN co-localization signal after naringin treatment (Figure 4D,E). Serum IL-1β and TNF-α were also elevated in DNP rats and were reduced after naringin administration (Figure 4F,G).

Phosphorylation of p38 MAPK and NF-κB p65 was increased in DRGs of DNP rats and was lower after naringin treatment (Figure 4H–K). ERK phosphorylation was likewise increased in DNP rats and reduced after naringin administration, whereas total ERK expression remained unchanged (Figure 4L,M). Collectively, these findings show that naringin treatment was accompanied by lower TRPV1 expression and attenuated MAPK (p38/ERK)-NF-κB-associated inflammatory signaling in DRGs. These molecular changes occurred alongside reduced systemic inflammatory markers and improved pain-related behaviors.

## 3. Discussion

The present study used complementary cellular and animal models to examine the effects of naringin on DNP-related phenotypes and associated molecular changes in DRG neurons. In HGHF-exposed primary DRG neurons, naringin attenuated loss of viability and apoptosis and was accompanied by lower TRPV1 expression, reduced intracellular Ca^2+^ accumulation, and decreased p38 MAPK/NF-κB activation. In the HGHF/low-dose STZ rat model, naringin reduced thermal and mechanical hypersensitivity, partially improved fasting blood glucose and body weight, lowered circulating IL-1β and TNF-α, and was associated with reduced TRPV1 expression and p38/ERK/NF-κB activation in DRGs. Together, these findings support a multi-target effect of naringin in experimental DNP while emphasizing that the measured molecular changes are associative rather than proof of a single causal pathway.

An important consideration is that the in vivo anti-nociceptive effects occurred together with improvements in metabolic parameters. Hyperglycemia and broader metabolic disturbances can contribute to DRG neuronal injury, inflammatory activation, and nociceptive sensitization. Analogous studies in other diabetes-associated complications have likewise shown that natural bioactive compounds can influence interconnected metabolic, oxidative, mitochondrial, inflammatory, and cell-death pathways [6,7]. The present design therefore cannot determine how much of the improvement in pain-related behavior and DRG molecular endpoints resulted from direct actions of naringin on sensory neurons versus indirect effects secondary to improved systemic metabolic control. The in vivo findings should accordingly be interpreted as potentially reflecting both direct neural actions and metabolic improvement. Future studies designed to dissociate glycemic effects from sensory-neuronal effects will be required to define their relative contributions.

TRPV1 is highly expressed in nociceptive sensory neurons and has been implicated in neuropathic pain [35,36]. Under diabetic conditions, altered TRPV1 expression or activity may be accompanied by increased Ca^2+^ influx and abnormal neuronal signaling [16,20,24]. In the present study, HGHF exposure increased TRPV1 expression and intracellular Ca^2+^, whereas naringin reduced both measures. Sustained Ca^2+^ dysregulation can contribute to mitochondrial dysfunction, caspase activation, and neuronal apoptosis [35], making the parallel changes in TRPV1 expression, Ca^2+^ signal, and neuronal survival biologically relevant. Nevertheless, TRPV1 activity was not manipulated directly, and the study did not use a selective TRPV1 antagonist, agonist, knockdown strategy, or other pathway-specific intervention. The data therefore support an association between naringin treatment and TRPV1-related Ca^2+^ dysregulation but do not demonstrate that TRPV1 is the causal mediator of the downstream cellular or behavioral effects.

The inflammatory findings should be interpreted in the same framework. Naringin reduced circulating IL-1β and TNF-α and was accompanied by lower phosphorylation of p38 MAPK, NF-κB p65, and ERK in DRGs. These pathways can interact with TRPV1 regulation and nociceptive sensitization [24,36,37,38], but their relationships are reciprocal and context dependent. Thus, the present results are more appropriately viewed as coordinated modulation of inflammatory signaling and TRPV1-related neuronal stress than as inhibition of a fixed TRPV1/Ca^2+^/p38/NF-κB cascade. Pathway-specific experiments will be needed to determine directionality, causal dependence, and the direct molecular target or targets of naringin.

Dose and exposure also require careful consideration. The cellular experiments examined 40 and 80 μM naringin after a broader preliminary viability screen, whereas the animal study evaluated only one oral dose (50 mg kg^−1^ day^−1^) and one 2-week treatment regimen. The absence of an in vivo dose–response or regimen comparison limits conclusions about the minimum effective dose, the exposure–response relationship, and whether efficacy or tolerability could be optimized by alternative dosing. In addition, orally administered naringin undergoes intestinal biotransformation to naringenin and other metabolites [32]. Because plasma pharmacokinetics and DRG tissue exposure were not measured, the present study cannot determine whether the observed in vivo effects were mediated by naringin itself, one or more metabolites, or indirect systemic mechanisms.

These considerations are particularly relevant to translation. Although the findings identify naringin as a compound of interest for further preclinical study, the 50 mg kg^−1^ rat dose cannot by itself define a clinically appropriate human dose or regimen. Translation will require pharmacokinetic and pharmacodynamic characterization, dose-ranging and safety studies, assessment of potential interactions with glucose-lowering and analgesic therapies, and confirmation that relevant exposures can be achieved in humans. Clinical DNP is also heterogeneous in duration, metabolic control, comorbidities, and pain phenotype, so efficacy in a single experimental model should not be assumed to generalize directly to patients.

Several additional limitations should be acknowledged. First, a formal prospective power calculation was not performed, and the final in vivo sample size was limited to six animals per group. Second, no osmotic control was included in the cellular experiments. Because the basal medium already contained 25 mM glucose and the HGHF condition contained 50 mM glucose plus FFAs, a contribution from increased extracellular osmolarity cannot be excluded. The in vitro model also represents a simplified metabolic-stress environment and does not reproduce the full cellular and vascular complexity of diabetic neuropathy. Third, the study used a single HGHF/low-dose STZ animal model and only male rats, which limits generalizability across experimental models and biological sex. Fourth, paw edema or paw thickness was not assessed because the primary behavioral objective was to characterize neuropathic nociceptive hypersensitivity rather than inflammatory paw swelling; such measurements could nevertheless provide complementary information in future studies. Likewise, the present study focused on TRPV1-related Ca^2+^ dysregulation, inflammatory cytokines, and MAPK/NF-κB-associated signaling and therefore did not include a broader oxidative/nitrosative stress panel (GSH, GST, catalase, MPO, and NO). Conventional histopathological assessment was also not performed; the immunofluorescence analysis provided neuronal localization of TRPV1 but does not substitute for structural neuropathology. Finally, the absence of pathway-specific interventions, an in vivo dose–response assessment, and pharmacokinetic/tissue-exposure measurements limits mechanistic and translational interpretation.

Overall, the data show that naringin attenuates DNP-related behavioral and cellular abnormalities and that these effects occur together with changes in metabolic status, inflammatory mediators, TRPV1 expression, intracellular Ca^2+^, and MAPK/NF-κB-associated signaling. The findings provide a rationale for more definitive studies incorporating larger prospectively powered cohorts, multiple DNP models, dose–response and pharmacokinetic analyses, and pathway-specific interventions.

## 4. Materials and Methods

### 4.1. Materials and Reagents

Primary rat DRG neurons were obtained from Shanghai Fuyu Biotechnology (Shanghai, China). Naringin was obtained from Aladdin (Shanghai, China; Lot G2017118), and streptozotocin (STZ; S8050), PBS, RIPA lysis buffer, PVDF membranes, and Triton X-100 were obtained from Solarbio (Beijing, China). The CCK-8 reagent was from Biochem (Shenzhen, China), the Annexin V-FITC/PI apoptosis detection kit was from Elabscience (Wuhan, China), the Estep Super Total RNA kit was from Promega (Shanghai, China), and EasyScript First-Strand cDNA Synthesis SuperMix and PerfectStart Green qPCR SuperMix were from TransGen Biotech (Beijing, China). Fluo-4 AM was from Beyotime (Shanghai, China). Primary antibodies included anti-TRPV1 (Bioss, Beijing, China), anti-p38 and anti-phospho-p38 (Cell Signaling Technology, Danvers, MA, USA), anti-NF-κB p65, anti-phospho-NF-κB p65, anti-ERK1/2 and anti-phospho-ERK1/2 (Proteintech, Wuhan, China), and anti-β-actin (Zhong Shan-Gold Bridge, Beijing, China); HRP-conjugated secondary antibodies were obtained from Proteintech. TRITC-conjugated goat anti-rabbit and FITC-conjugated goat anti-mouse secondary antibodies used for immunofluorescence were from Zhong Shan-Gold Bridge. Serum TNF-α and IL-1β ELISA kits were from R&D Systems (Minneapolis, MN, USA; catalog numbers MTA00B and RLB00, respectively). Other routine cell-culture and biochemical reagents included high-glucose DMEM, fetal bovine serum, penicillin/streptomycin, D-glucose, oleic acid, palmitic acid, DMSO, sodium pentobarbital, donkey serum, NeuN antibody, DAPI, and antifade mounting medium.

### 4.2. Cell Culture and Experimental Treatments

Primary rat dorsal root ganglion (DRG) neurons (Shanghai Fuyu Biotechnology, Shanghai, Chin) were cultured in high-glucose Dulbecco’s Modified Eagle Medium (DMEM) containing 25 mM D-glucose and supplemented with 10% fetal bovine serum, 100 U mL^−1^ penicillin, and 100 μg mL^−1^ streptomycin. Thus, the 25 mM glucose concentration used for the control condition reflected the glucose concentration of the basal high-glucose DMEM rather than a separately selected physiological glucose concentration. For the HGHF condition, additional D-glucose was added to achieve a final glucose concentration of 50 mM together with 0.60 mM free fatty acids (FFAs). The FFA mixture consisted of oleic acid and palmitic acid at a 2:1 (*w*/*w*) ratio (equivalent to palmitate:oleate = 1:2). A 200 mM FFA stock was prepared by dissolving 36 mg oleic acid and 18 mg palmitic acid in 1 mL DMSO with ultrasonic water-bath assistance; 6 μL of this stock was added to 2 mL culture medium per well to obtain a final FFA concentration of 0.60 mM. No separate osmotic control was included in the present experiments.

After overnight serum deprivation, neurons were exposed for 48 h to the following conditions: Control (25 mM glucose), HGHF (50 mM glucose + 0.60 mM FFAs), NG40 (HGHF + 40 μM naringin), or NG80 (HGHF + 80 μM naringin). A preliminary naringin concentration screen (40–200 μM) was used to define the non-cytotoxic range. Concentrations of 40 and 80 μM were selected for subsequent experiments as lower and higher concentrations within this range; 80 μM was retained because it showed a more evident protective effect than 40 μM in the preliminary cellular assessment and was consistent with the concentration used in previous work from our laboratory.

### 4.3. Cell Viability and Apoptosis

Cell viability was quantified using the Cell Counting Kit-8 (CCK-8; Biochem, Shenzhen, China). DRG neurons were plated at 5 × 10^4^ cells per well in 96-well plates. After 48 h of treatment, 10 μL CCK-8 reagent was added to each well, followed by incubation at 37 °C for 1 h. Absorbance at 450 nm was measured using a SpectraMax i3x multimode microplate reader (Molecular Devices, San Jose, CA, USA), supplied by Danaher (Shanghai) Enterprise Management Co., Ltd. (Shanghai, China). Each independent experiment included at least five technical wells per treatment condition, and the experiments were repeated independently three times.

Apoptosis was assessed using an Annexin V-FITC/propidium iodide (PI) apoptosis detection kit (Elabscience, Wuhan, China) according to the manufacturer’s instructions. DRG neurons were plated at 5 × 10^4^ cells per well in 6-well plates, treated for 48 h, harvested, washed with cold PBS, resuspended in binding buffer, and incubated with Annexin V-FITC and PI for 10–15 min in the dark at room temperature. Samples were immediately analyzed using a FACSCalibur flow cytometer (BD Biosciences, San Jose, CA, USA). Three independent experiments were performed.

### 4.4. Quantitative Real-Time PCR (qRT-PCR)

For in vitro analyses, cultured DRG neurons were collected after the indicated treatments. For in vivo analyses, harvested L4–L6 DRGs were rinsed in ice-cold PBS (Solarbio, Beijing, China), snap-frozen in RNA storage solution, and stored at −80 °C until processing. Total RNA was isolated using the Estep Super Total RNA kit (Promega, Shanghai, China), and first-strand cDNA was synthesized using EasyScript First-Strand cDNA Synthesis SuperMix (TransGen Biotech, Beijing, China). qRT-PCR was performed on a CFX96 Real-Time System (Bio-Rad, Hercules, CA, USA) using PerfectStart Green qPCR SuperMix (TransGen Biotech) and gene-specific primers. Relative TRPV1 mRNA expression was determined using the 2^−ΔΔCt^ method, with β-actin as the internal reference gene and the corresponding Control group as the calibrator. Each sample was analyzed in technical triplicate, and the mean Ct value of the three technical replicates was used for subsequent calculation. ΔCt was calculated as Ct(TRPV1) − Ct(β-actin), and ΔΔCt was calculated relative to the Control group. Primer amplification efficiency was evaluated using standard curves generated from serially diluted cDNA and was within the acceptable range of 90–110%, with correlation coefficients (R^2^) ≥ 0.99. Amplification specificity was confirmed by single-peak melting-curve analysis. Relative expression is presented as 2^−ΔΔCt^ fold change, whereas statistical comparisons were performed using ΔCt values.

Primer sequences were as follows: TRPV1 forward, 5′-ATGACACCATCGCTCTGCTC-3′; TRPV1 reverse, 5′-GTGCTGTCTGGCCCTTGTAG-3′; β-actin forward, 5′-CCTAAGGCCAACCGTGAAAAGA-3′; β-actin reverse, 5′-GGTACGACCAGAGGCATACA-3′.

### 4.5. Western Blotting

For in vitro experiments, DRG neurons were lysed after the indicated treatments, and three independent culture experiments were analyzed. For in vivo experiments, L4–L6 DRGs were dissected, rinsed in ice-cold PBS, and stored at −80 °C before protein extraction. Two DRGs from the same animal were pooled to generate one animal-level lysate; tissues from different animals were not pooled. For in vivo Western blotting, three animals per group were analyzed, with each animal-level lysate representing one independent biological replicate (*n* = 3 biological replicates per group). Cell or tissue samples were lysed/homogenized in ice-cold RIPA buffer (Solarbio) supplemented with 1% protease and phosphatase inhibitor cocktail, incubated on ice for 20 min, and centrifuged at 14,000× *g* rpm for 15 min at 4 °C. Supernatants were mixed with 6× loading buffer (1:5, *v*/*v*), boiled for 5 min, and stored at −20 °C until analysis.

Proteins were separated on 10% SDS-PAGE gels and transferred to PVDF membranes (Solarbio). Membranes were incubated overnight at 4 °C with primary antibodies against TRPV1 (rabbit, 1:1000, Bioss), p38 (rabbit, 1:1000, Cell Signaling Technology), phospho-p38 (rabbit, 1:500, Cell Signaling Technology), NF-κB p65 (1:1000, Proteintech, Wuhan, China), phospho-NF-κB p65 (1:1000, Proteintech, Wuhan, China), ERK1/2 (1:1000, Proteintech, Wuhan, China), phospho-ERK1/2 (1:1000, Proteintech, Wuhan, China), and β-actin (mouse, 1:1000, Zhong Shan-Gold Bridge). After washing, membranes were incubated with HRP-conjugated secondary antibodies (1:2000, Proteintech) for 2 h at room temperature. Immunoreactive bands were visualized using a ChemiDoc XRS+ system (Bio-Rad) and quantified using quantified using ImageJ 1.47. TRPV1 was normalized to β-actin, whereas phosphorylated proteins were normalized to their corresponding total proteins.

### 4.6. Intracellular Ca^2+^ Imaging

DRG neurons were loaded with Fluo-4 AM (Beyotime, Shanghai, China), a fluorescent Ca^2+^ indicator widely used for cellular Ca^2+^ imaging [39]. After three PBS washes to remove serum and phenol red, a 1× Fluo-4 AM working solution prepared in assay buffer was added, and cells were incubated at 37 °C for 30 min in the dark. After a final PBS rinse, fluorescence (Ex/Em = 490/525 nm) was immediately captured using an inverted fluorescence microscope under identical acquisition settings across treatment groups. Ca^2+^ imaging was performed in three independent culture experiments. At least five non-overlapping microscopic fields were examined in each independent experiment, with a total of 50 neurons analyzed per treatment group across the three experiments. Individual neurons within the same culture experiment were treated as observational subsamples; fluorescence measurements were summarized at the experiment level, and the independent culture experiment served as the statistical unit.

### 4.7. Animal Model, Experimental Groups, and Naringin Administration

Male Sprague–Dawley rats (180–200 g; Changsha Tianqin Biotechnology, Changsha, China) were housed under standard laboratory conditions (22 ± 2 °C, with free access to food and water). All procedures were approved by the Ethics Committee of Jiangxi Medical College (Approval No. GYZFY2023012) and were conducted in accordance with institutional guidelines. Thirty-five rats entered the protocol. Six rats assigned to the Control group were maintained on standard chow throughout the study, whereas 29 rats entered the model-induction protocol. Model animals were fed an HGHF diet (SYHF45-1; Anhui Kuibu Shuyu Biotechnology Co., Ltd., Ningguo, China) for 4 weeks before STZ injection and remained on the same diet thereafter. The diet contained, by weight, 43.6% basic diet, 17.5% lard, 12.0% sucrose, 10.0% whole-milk powder, 10.0% casein, 2.0% mineral/vitamin mixture, 1.9% agar, 2.0% calcium hydrogen phosphate, and 1.0% calcium carbonate, and was formulated as an approximately 45% fat-energy diet.

The HGHF/low-dose STZ model of DNP was established with reference to our previous experimental protocol, with modifications [24]. After the 4-week dietary period, model animals were fasted overnight and given a single intraperitoneal injection of STZ (35 mg kg^−1^; S8050, Solarbio). Diabetes was defined 72 h later as fasting blood glucose ≥ 7.8 mmol L^−1^ or random blood glucose ≥ 16.7 mmol L^−1^. In diabetic animals, DNP was considered established when both MWT and TWL decreased to <80% of the corresponding baseline values within 14 days after STZ administration. Three animals died within 72 h after STZ, possibly because of acute STZ toxicity or early hypoglycemia. Twenty-six animals met the diabetes criterion, of which 22 met the DNP criterion; four diabetic rats that did not show the required reduction in MWT and TWL were excluded. The 22 DNP-positive animals were subsequently randomized into the three model/treatment groups before gavage (DNP, *n* = 8; Naringin, *n* = 7; Vehicle, *n* = 7). During the gavage period, two rats in the DNP group and one rat each in the Naringin and Vehicle groups died, possibly in association with severe hyperglycemia and diabetic complications, leaving six animals in each group for final analysis. No formal prospective power calculation was performed; the target sample size was based on previous studies using comparable DNP models, preliminary observations, and the 3Rs principle, and the relatively small sample size is acknowledged as a limitation.

After diabetes and DNP were confirmed, eligible rats were allocated to the DNP, Naringin, and Vehicle groups using a random-number table. The randomization sequence was generated and retained by an investigator who was not involved in behavioral testing, drug administration, or data analysis, and group allocation was concealed until treatment began. Investigators performing behavioral testing, tissue collection, image acquisition, and quantitative Western blot/immunofluorescence analyses were blinded to treatment allocation. The investigator responsible for gavage was aware of group allocation but did not participate in behavioral testing or molecular data analysis; blinding was maintained until quantitative analyses were completed. The DNP group received no treatment, the Vehicle group received 5% DMSO in saline, and the Naringin group received naringin. The Vehicle group was used as the primary comparator for the treatment effect of naringin, whereas the Control group was used to characterize disease-associated changes.

Naringin (Aladdin; Shanghai, China, Lot G2017118) was dissolved in 5% DMSO/saline with brief warming to prepare a 10% (*w*/*v*) stock and was further diluted for dosing. The 50 mg kg^−1^ day^−1^ dose was selected with reference to previously reported oral naringin doses spanning 10–100 mg kg^−1^ day^−1^ in related diabetic neuropathy models. Beginning at the end of week 6, immediately after the pretreatment behavioral assessment, rats received daily intragastric administration of naringin or vehicle for 2 weeks. At week 8, rats were deeply anesthetized with sodium pentobarbital (50 mg kg^−1^, intraperitoneally) and euthanized by exsanguination followed by cervical dislocation. Death was confirmed by the absence of heartbeat and respiration. Bilateral L4–L6 DRGs were rapidly harvested for molecular analyses, and tissues from age-matched control rats were processed in parallel.

### 4.8. Mechanical Withdrawal Threshold

Mechanical nociceptive sensitivity was assessed using an electronic mechanical analgesia tester (BME-404, Tianjin, China; pressure range 0–50 g). Rats were placed on a metal mesh platform and allowed to remain quiet before testing. Mechanical stimulation was applied to the left hind paw, and the force (g) eliciting a clear paw-withdrawal or licking response was recorded as the mechanical withdrawal threshold (MWT). Three measurements were obtained for each animal at each assessment and averaged to generate one animal-level value. MWT was assessed at weeks 0, 1, 5, 6, 7, and 8.

### 4.9. Thermal Withdrawal Latency

Thermal nociceptive sensitivity was assessed using a plantar analgesia tester (BME-410C, Tianjin, China). Rats were placed individually in transparent acrylic enclosures and allowed to settle before testing. Thermal stimulation was applied to the left hind paw with a 30 s cutoff, and the latency to paw withdrawal or licking was recorded as the thermal withdrawal latency (TWL). Group data at each time point comprised the six animals in each group. TWL was assessed at weeks 0, 1, 5, 6, 7, and 8. Fasting blood glucose and body weight were monitored on days 0, 3, 7, 14, 17, and 21 beginning at week 5, corresponding to repeated assessments across weeks 5–8.

### 4.10. Immunofluorescence Staining

L4–L6 DRGs were rinsed in ice-cold PBS and fixed in 4% paraformaldehyde for 2 h. After overnight cryoprotection in 20% sucrose at 4 °C, ganglia were embedded in optimal-cutting-temperature compound and serially sectioned at 10 μm on a cryostat. Sections were air-dried for 3 min, washed three times in PBS (5 min each), permeabilized with 0.3% Triton X-100 (Solarbio, Beijing, China) for 15 min, and blocked with 10% donkey serum for 1 h at 37 °C. Sections were incubated overnight at 4 °C with rabbit anti-TRPV1 (1:100) and mouse anti-NeuN (1:100, Abcam, Cambridge, UK), followed by TRITC-conjugated goat anti-rabbit and FITC-conjugated goat anti-mouse secondary antibodies (1:200; Zhong Shan-Gold Bridge) for 1 h at room temperature. Nuclei were counterstained with DAPI, and sections were mounted with antifade medium and imaged using an Olympus FV1200 confocal microscope (Tokyo, Japan). DRGs from 3–4 animals per group were analyzed; four sections per animal, separated by approximately 7–8 μm, were included in the immunofluorescence assessment. Three non-overlapping fields were analyzed per section. Fields were selected from comparable regions of the DRG while avoiding tissue edges, folds, damaged areas, and obvious sectioning artifacts, and field selection and image analysis were performed by investigators blinded to group allocation. All images used for quantitative comparison were acquired using identical microscope settings, including laser power, detector gain, pinhole, magnification, and image resolution. TRPV1/NeuN co-localization was quantified using ImageJ 1.47 with the Coloc 2 plugin. A consistent automated thresholding procedure was applied across all images, and Pearson’s correlation coefficient was used as the co-localization metric. Measurements from individual fields and sections were first averaged within each animal, and the animal, rather than individual fields or sections, served as the biological and statistical unit.

### 4.11. Enzyme-Linked Immunosorbent Assay (ELISA)

Serum TNF-α and IL-1β were quantified using commercially available ELISA kits (TNF-α, MTA00B; IL-1β, RLB00; R&D Systems) according to the manufacturer’s protocol. Blood was collected by cardiac puncture, centrifuged at 1000× *g* for 15 min at 4 °C, and serum aliquots were stored at −80 °C until analysis. Samples were diluted as recommended, and standards were serially diluted to generate calibration curves. Optical density was measured at 450 nm using a microplate reader, and cytokine concentrations were calculated from the corresponding standard curves.

### 4.12. Statistical Analysis

Data were graphed and statistically analyzed using GraphPad Prism 8.4.0 (GraphPad Software, San Diego, CA, USA) and IBM SPSS Statistics 21 (IBM, Chicago, IL, USA). Body weight, fasting blood glucose (FBG), thermal withdrawal latency (TWL), and mechanical withdrawal threshold (MWT), which were repeatedly measured in the same animals, were analyzed using two-way repeated-measures ANOVA with treatment group as the between-subject factor and time as the within-subject factor. Main effects of group and time and the group × time interaction were evaluated. Sphericity was assessed using Mauchly’s test. When the assumption of sphericity was violated, Greenhouse–Geisser correction was applied. Significant group × time interactions were followed by simple-effects analyses with Bonferroni-adjusted pairwise comparisons. Independent end-point data involving more than two groups were analyzed using one-way ANOVA with Bonferroni-adjusted post hoc comparisons. The Vehicle group served as the primary comparator for the naringin treatment effect in vivo, while Control comparisons were used to describe disease-associated changes. For qRT-PCR data, statistical comparisons were performed using ΔCt values, whereas relative expression is presented as 2^−ΔΔCt^ fold change for graphical presentation. For Ca^2+^ imaging, the independent culture experiment, rather than individual neurons, served as the statistical unit. Data are presented as mean ± SD, and *p* < 0.05 was considered statistically significant.

## 5. Conclusions

Using primary DRG neurons exposed to HGHF metabolic stress and an HGHF/low-dose STZ rat model, this study found that naringin attenuated neuronal injury, thermal and mechanical hypersensitivity, systemic inflammatory markers, TRPV1 expression, intracellular Ca^2+^ dysregulation, and MAPK/NF-κB-associated signaling changes. The in vivo effects occurred together with partial improvement in fasting blood glucose and body weight, so a contribution from improved metabolic control cannot be separated from direct neural actions. The molecular findings demonstrate coordinated associations rather than a causal or strictly linear TRPV1/Ca^2+^/MAPK/NF-κB cascade. The principal limitations include the small animal cohort without prospective power calculation, absence of pathway-specific interventions and an osmotic control, evaluation of a single in vivo dose and animal model, and lack of pharmacokinetic or tissue-exposure measurements. Future studies should incorporate dose-ranging and exposure–response analyses, additional DNP models and phenotypic endpoints, and selective mechanistic interventions before the translational potential of naringin can be defined.

## Data Availability

The data supporting the findings of this study are available from the corresponding author upon request.
